# Optimizing Low Fishmeal Diets with Vitamin C Supplementation: A Comprehensive Study on Growth, Immunity, and Heat Stress Resistance in Largemouth Bass (*Micropterus salmoides*) Juveniles

**DOI:** 10.3390/antiox14101175

**Published:** 2025-09-26

**Authors:** Shengqi Zhao, Hualiang Liang, Xiaoru Chen, Lu Zhang, Dongyu Huang, Yongli Wang, Zhenyan Cheng, Mingchun Ren

**Affiliations:** 1 College of Fisheries, Tianjin Agricultural University, Tianjin 300392, China; 17622738771@163.com; 2 Key Laboratory of Integrated Rice-Fish Farming Ecology, Ministry of Agriculture and Rural Affairs, Freshwater Fisheries Research Center, Chinese Academy of Fishery Sciences, Wuxi 214081, China; lianghualiang@ffrc.cn (H.L.); huangdongyu@ffrc.cn (D.H.); 3 Healthy Aquaculture Key Laboratory of Sichuan Province, Tongwei Co., Ltd., 588 Tianfu Avenue, Chengdu 610093, China; chenxr@tongwei.com (X.C.); zhangl21@tongwei.com (L.Z.); wangyl@tongwei.com (Y.W.)

**Keywords:** low fishmeal, vitamin C, farmed largemouth bass, immunity response, heat stress

## Abstract

Six dietary groups were supplemented with graded vitamin C (VC) levels: VC1 (control, 0.39 g/kg), VC2 (0.51 g/kg), VC3 (0.66 g/kg), VC4 (0.81 g/kg), VC5 (0.97 g/kg), and VC6 (1.11 g/kg). Largemouth bass (*Micropterus salmoides*) with an initial weight of 2.21 ± 0.00 g were fed these diets for 8 weeks to evaluate the effects of different VC levels on growth performance, immune response, and heat stress resistance. Heat stress was induced at a constant temperature of 33.00 ± 0.16 °C for one week. The VC3 and VC4 groups showed significantly improved growth performance (FBW, WGR, SGR) compared to VC1 (*p* < 0.05). VC4 exhibited lower ALT and AST levels before and after heat stress. Antioxidant capacity (T-AOC, GSH-Px, CAT) was significantly enhanced in VC3–VC5, with VC5 showing the highest after stress activity (except CAT). Expression of pro-inflammatory genes (*nf-κb*, *il-8*) was downregulated in VC4 and VC5, while anti-inflammatory *il-10* was upregulated in VC4 after stress. Apoptosis-related genes (*bcl-2*, *caspase*, *bax*) and TUNEL assays indicated the strongest anti-apoptotic effects in VC3 and VC4 under heat stress (*p* < 0.05). These findings suggest that VC supplementation in low-fishmeal diets enhances growth, immune response, apoptosis resistance, and acute heat stress tolerance in fish.

## 1. Introduction

Fishmeal, as the primary protein source in aquaculture diets, is rich in protein, essential amino acids, vitamins, minerals, and other necessary nutrients [1]. Nevertheless, alongside the swift advancement of socioeconomic development, the aquaculture industry has grown significantly, leading to a mismatch between the demand for fishmeal and its availability, resulting in rising fishmeal prices and limiting the growth of the industry. To ensure the sustainable development of aquaculture, alleviate resource pressures, reduce feed costs, and promote environmentally friendly practices, low-fishmeal diets have been increasingly adopted. Despite this, research has shown that low-fishmeal diets can negatively affect the physiological metabolism of farmed species [2,3,4,5]. The supplementation of additives such as enzymes [6], probiotics and prebiotics [7], natural plant extracts [8], and Vitamin C (VC) [9] can enhance the performance of aquatic animals fed low-fishmeal diets. Therefore, investigating the effects of specific additives in low-fishmeal diets is essential for optimizing aquaculture practices.

VC, or L-ascorbic acid, is essential for the normal growth and physiological functions of aquatic animals [10]. VC plays a critical role in promoting fish growth [11], supporting reproductive health [12], enhancing iron absorption [13], strengthening immunity [13], improving tissue hematology [14], and facilitating collagen synthesis [15]. Moreover, VC acts as a potent antioxidant by donating electrons to biomolecules, protecting cells from oxidative damage during metabolism [16], which is largely attributed to its influence on the Nrf2 signaling pathway [17]. Supplementing 148 mg/kg VC in a low-fishmeal diet for hybrid grouper (*Epinephelus fuscoguttatus* ♀ × *Epinephelus lanceolatus* ♂) enhances the Nrf2 pathway, thereby boosting antioxidant capacity [9]. Additionally, VC supplementation has been effective in reducing lipid peroxide accumulation and improving the growth performance of tuna (*Terapon jarbua*) [18,19]. However, limited research has explored the effects of VC supplementation in low-fishmeal diets within aquaculture, highlighting a significant gap that warrants further investigation.

Recent reports indicate that the total production of *Micropterus salmoides* (*M. salmoides*) in China has reached 880,000 tonnes, accounting for over 99% of global production [20,21]. This species is highly valued for its meat quality, rapid growth, and adaptability [22,23]. However, high water temperatures during summer result in elevated mortality rates in *M. salmoides* [24]. VC supplementation in the diet can effectively enhance growth rate [25], immunity [26], and antioxidant capacity in *M. salmoides* [27]. As previously noted, low-fishmeal diets may lead to nutritional deficiencies, compromised immune function, and reduced stress tolerance in fish. Given VC’s ability to improve growth and immune responses, this study aims to evaluate the impact of VC supplementation in low-fishmeal diets on the growth, immunity, and stress resilience of *M. salmoides*. The study will also determine the optimal VC level in these diets. Additionally, heat stress conditions were incorporated to assess how VC supplementation affects the alleviation of acute heat stress in *M. salmoides* under low-fishmeal diet conditions.

## 2. Materials and Methods

### 2.1. Diet Preparation

Table 1 presents the primary components and dry matter content of the experimental diets. These diets primarily consist of fishmeal and chicken meal as the primary protein constituents, supplemented by fish oil and soybean oil as the principal fat inputs, among others. By reviewing relevant studies on vitamin C in aquaculture fish, based on a review of studies on VC in aquaculture fish species, optimal supplementation levels vary significantly among different animals, as reported in previous research [28,29,30,31,32,33]. Six diets with varying VC concentrations were formulated, named VC1, VC2, VC3, VC4, VC5, and VC6, corresponding to VC levels of 0.39 g/kg, 0.51 g/kg, 0.66 g/kg, 0.81 g/kg, 0.97 g/kg, and 1.11 g/kg, respectively. All ingredients were milled, passed through an 80-mesh sieve, accurately weighed, and mixed with water according to the designed formulations [34]. The mixture was then processed into puffed pellets (3 mm in diameter) using a bulking granulator (TSE65-type, Beijing, China) as described in previous studies. After pellet formation, the diets were naturally air-dried at ambient temperature. They were then placed in resealable bags, marked, and preserved at −20 °C for later use.

### 2.2. Experimental Fish

Juvenile *M. salmoides* were cultured in the recirculating aquaculture system (RAS) at the Freshwater Fisheries Research Centre of the Chinese Academy of Fisheries Sciences (CAFS), located in Yixing, China. Prior to the experiment, all juvenile fish were acclimated for two weeks in RAS breeding buckets (270 L) and fed a commercial compound diet (48% protein, 12% lipid) to adapt to the culture environment. A total of 360 healthy, uniformly sized juvenile fish (initial weight 2.21 ± 0.00 g) were randomly selected and distributed into RAS breeding buckets (20 fish per bucket), with 18 buckets set up to represent the six dietary treatments, each with triplicates. The fish received three daily feedings at 07:30, 12:30, and 17:30, with each meal provided until satiation. The culture period lasted for 8 weeks. Water quality parameters (YSI ProDSS Multiparameter Water Quality Meter, OH, USA) were recorded daily, with water temperature maintained between 26 and 30 °C, dissolved oxygen concentration above 6 mg/L, and pH between 7.0 and 7.5.

### 2.3. Sample Collection

When the farming trial concluded, the fish were maintained without feeding for 24 h, after which the fish in each bucket were weighed and counted for growth performance analysis. Blood was collected from the tail vein of three randomly selected fish from each bucket, placed in centrifuge tubes, and immediately centrifuged (10 min, 3000 rpm, 4 °C). The supernatant was transferred into corresponding tubes and stored at −80 °C for subsequent plasma biochemical and antioxidant capacity analyses. Intestinal samples were taken for gene expression analysis related to antioxidants, immunity, and apoptosis. Additionally, two more fish from each bucket were randomly selected for whole-body composition analysis.

### 2.4. Heat Stress Experiment

At the end of the previous culture period, the remaining fish were placed back into the same RAS breeding buckets, maintaining the same groupings as before. The water temperature was incrementally raised from 28 °C to 33 °C at a rate of 0.5 °C per hour using a heating rod. The temperature was monitored every three hours with a thermometer until it stabilized at 33 ± 0.16 °C. During this period, the culture environment was maintained under the same conditions as the previous phase, with the dissolved oxygen concentration kept above 6 mg/L and the pH maintained between 7.0 and 7.5. The fish were cultured for an additional week under these conditions. Sample collection after the heat stress period was conducted following the methods outlined in Section 2.3. In addition to the previously collected samples, gills from each fish were harvested for TUNEL immunofluorescence analysis.

### 2.5. Measurement of Growth Indicators

The main growth indicators were calculated according to the specific formulae in Table 2.

### 2.6. Body Composition and Biochemical Analysis

The whole fish body composition and the crude composition of the feed were determined using methods referenced from previous studies within our group. Firstly whole fish moisture (%) was determined by drying in an oven at 105 °C. Whole fish and feed crude protein (%) were determined using a Kjeldahl nitrogen meter (Haineng K1100, Jinan Haineng Instrument Co., Ltd., Jinan, China). Whole fish and feed crude lipid (%) were determined on an automatic analyser (Haineng SOX606, Jinan Haineng Instrument Co., Ltd., China) using the Soxhlet extraction method. The ash (%) content of whole fish and feed was determined by burning in a muffle furnace (XL-2A, Hangzhou Zhuochi Instrument Co., Ltd., Hangzhou, China) at 560 °C. Plasma biochemical parameters ALT (alanine aminotransferase) and AST (aspartate aminotransferase) were measured using the methods recommended by the International Federation of Clinical Chemistry, employing kits purchased from Mindray Medical International Ltd. (Shenzhen, China) (ALT: 105-000442-00; AST: 15-00443-000) on the Mindray BS 400 automated biochemical analyser (Mindray Medical International Ltd., Shenzhen, China), as described in the experimental protocols of previous research [35]. Plasma antioxidant enzyme activity factors MDA (malondialdehyde), CAT (catalase), SOD (superoxide dismutase), GSH-Px (glutathione peroxidase), GSH (glutathione) and T-AOC (total antioxidant capacity) were measured using assay kits purchased from Jiancheng Bioengineering Institute (Nanjing, China). MDA was determined using the TBA method for amino acids (A003-1-2), CAT was measured by the molybdate method (A007-1-1), SOD was assessed via the WST-1 assay (A001-3-2), GSH was quantified using the microplate method (A006-1-1), GSH-Px was determined by the colorimetric method (A005-1-2), and T-AOC was measured by the ABTS method (A015-2-1). The primary experimental methods, kits, and equipment followed those detailed in the earlier research [35], as outlined in Table 3.

### 2.7. Methods of Analysing Apoptosis in Gills After Heat Stress

The analysis was conducted using the Aipathwell image analysis software (Alpathwell v2) from Servicebio. The procedure involved the following steps: (1) Positioning: The area to be measured was selected either automatically or manually. (2) Color Selection: The target fluorescence signal in HSI was manually selected to ensure accurate identification of the positive signal, which was then saved as a standard. (3) Calculation: The software automatically identifies DAPI-stained cell nuclei, extends the cytoplasmic boundaries, and computes the number of positive cells, area, cumulative optical density (IOD), and tissue area after reverse decolorization. (4) Analysis: The software processes high-power data of the measured area and generates a report.

### 2.8. Real-Time PCR Analysis

Following the method outlined by D. Huang et al. [35], RNA was extracted from perch intestines using the TRIzol method (Vazyme Biotech Co., Ltd., Nanjing, China). RNA concentration and purity were determined using a NanoDrop 2000 spectrophotometer. Real-time quantitative PCR analysis of genes related to immunity, antioxidant capacity, and apoptosis was performed on a CFX96 real-time PCR detection system (Bio-Rad) following the kit protocol (Q221-01, Vazyme, Nanjing, China). Beta-actin (*β-actin*) was selected as the internal reference gene due to its stability in prior studies within the group. Primer sequences are provided in Table 4.

### 2.9. Statistical Analyses

Growth performance data and whole fish body composition are presented as Mean ± Standard Error (Mean ± S.E.) and analyzed using one-way analysis of variance (ANOVA) with Tukey’s method in SPSS ver. 26 to determine statistical significance (*p* < 0.05). Independent *t*-tests were used for comparisons before and after heat stress, with *p* < 0.05 indicating statistical significance and *p* < 0.01 denoting high significance. Graphs and tables were created using GraphPad Prism software (GraphPad Prism version 9.1.0 for Windows (GraphPad Software, San Diego, CA, USA).

## 3. Results

### 3.1. Growth Data

**Figure 1:**
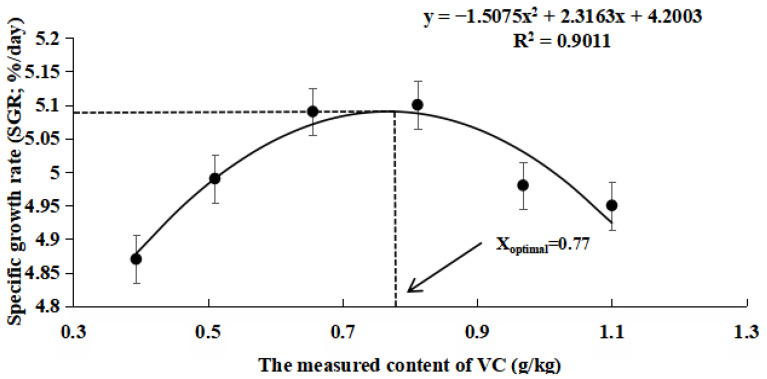
Quadratic regression relationship between specific growth rate (SGR,%/day) and VC supplementation levels in low-fishmeal diets.

**Figure 2:**
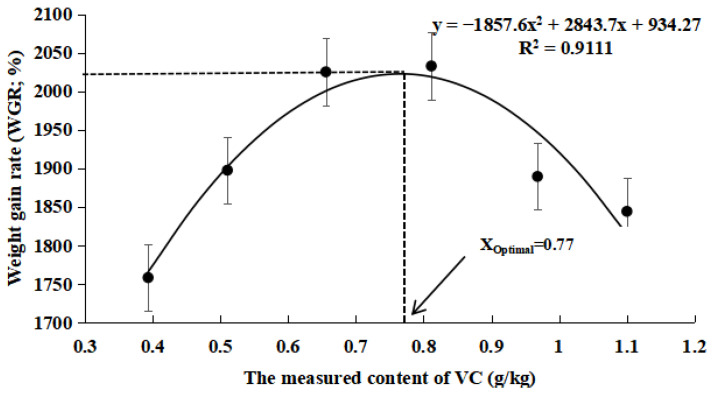
Quadratic regression relationship between weight gain rate (WGR,%) and VC supplementation levels in low-fishmeal diets.

As shown in Table 5, the FBW, WGR, and SGR were significantly higher in the VC3 and VC4 groups compared to the VC1 group (*p* < 0.05). The FCR decreased markedly in the VC2, VC5, and VC6 groups compared to the VC1 group (*p* < 0.05). Quadratic regression analysis of SGR (Figure 1) and WGR (Figure 2) with varying VC levels indicated that the optimal VC requirement for the diet was 0.77 g/kg. According to Table 6, different VC concentrations in the diet did not significantly affect the whole-body composition of *M. salmoides* (*p* > 0.05).

### 3.2. Plasma Biochemical Analysis Before and After Heat Stress

**Figure 3:**
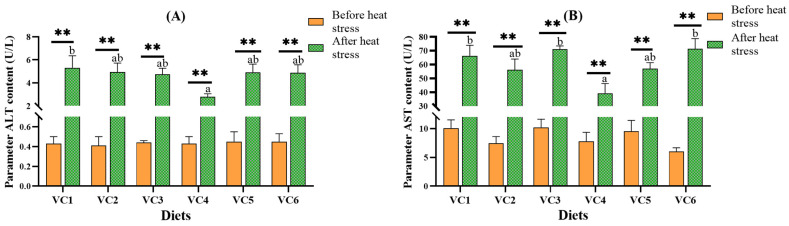
Effect of different levels of VC added to low fishmeal diets on plasma biochemical parameters of *M. salmoides* before and after heat stress. Data after heat stress are marked with lowercase letters ‘a–b’. After *t*-test, ‘**’ for before and after heat stress data shows a extremely significant contrast (*p* < 0.01). Columns of identical color marked with different letters show significant variations between data points (*p* < 0.05). (**A**), ALT; (**B**), AST.

Figure 3 illustrates the effect of dietary VC levels on plasma biochemical indices of *M. salmoides* before and after heat stress. No significant impact on ALT and AST concentrations was observed in all groups before heat stress (*p* > 0.05). After heat stress, both ALT and AST levels in the VC4 group were markedly lower than those in the VC1 group (*p* < 0.05). *t*-test analysis revealed significant increases in ALT and AST levels after heat stress across all groups (*p* < 0.01).

### 3.3. Analysis of Plasma Antioxidant Capacity

**Figure 4:**
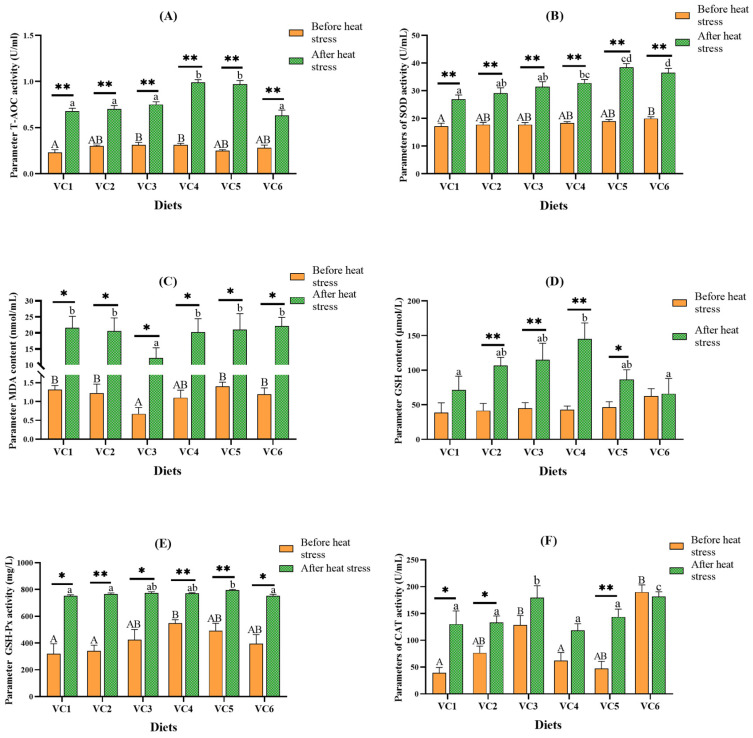
Effect of different levels of VC added to low fishmeal diets on plasma antioxidant capacity of *M. salmoides* before and after heat stress. Data before heat stress are marked with uppercase letters ‘A–B’ and data after heat stress are marked with lowercase letters ‘a–d’. After *t*-test, ‘*’ for before and after heat stress data shows a statistically significant contrast (*p* < 0.05) and ‘**’ shows extremely significant (*p* < 0.01). Columns of identical color marked with different letters show significant variations between data points (*p* < 0.05). (**A**), T-AOC; (**B**), SOD; (**C**), MDA; (**D**), GSH; (**E**), GSH-Px; (**F**), CAT.

Figure 4 presents the effects of different VC levels in the diet on the plasma antioxidant capacity of *M. salmoides* before and after heat stress. Before stress, the activity of T-AOC (Figure 4A) was significantly higher in the VC3 and VC4 groups compared to the VC1 group (*p* < 0.05). SOD activity (Figure 4B) was significantly increased in the VC6 group (*p* < 0.05). MDA content (Figure 4C) was lowest in the VC3 group (*p* < 0.05). CAT activity (Figure 4E) was markedly higher in the VC3 and VC6 groups than in the VC1 group (*p* < 0.05). GSH-Px activity (Figure 4E) was significantly higher in the VC4 group than in the VC1 group (*p* < 0.05). After heat stress, the activities of T-AOC (Figure 4A), SOD (Figure 4B), and GSH-Px (Figure 4E) were considerably higher in the VC5 group compared to the VC1 group (*p* < 0.05). MDA content (Figure 4C) remained lowest in the VC3 group (*p* < 0.05), while GSH levels (Figure 4D) were significantly higher in the VC4 group (*p* < 0.05). The highest CAT activity (Figure 4E) was observed in the VC3 and VC6 groups (*p* < 0.05). *t*-test analysis revealed that the activities of T-AOC and SOD were significantly higher after heat stress compared to before heat stress (*p* < 0.05), with GSH levels significantly elevated in the VC2-VC5 groups.

### 3.4. Analysis of Antioxidant-Related Genes in the Intestine

**Figure 5:**
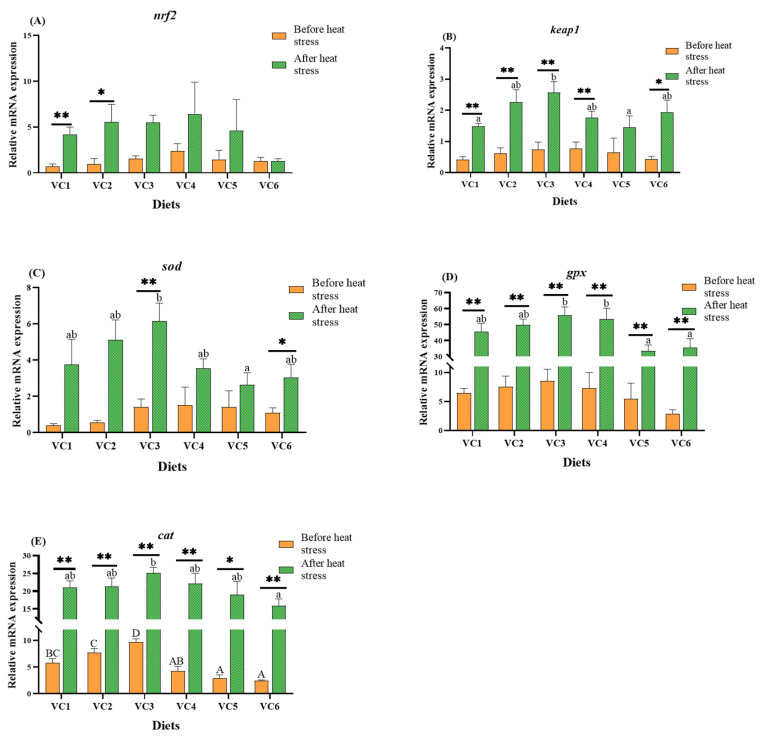
Effects of different levels of VC added to low fishmeal diets on antioxidant-related genes in the intestine of *M. salmoides* before and after heat stress. Data before heat stress are marked with uppercase letters ‘A–D’ and data after heat stress are marked with lowercase letters ‘a–b’. After *t*-test, ‘*’ for before and after heat stress data shows a statistically significant contrast (*p* < 0.05) and ‘**’ shows extremely significant (*p* < 0.01). Columns of identical color marked with different letters show significant variations between data points (*p* < 0.05). (**A**), *nrf2*; (**B**), *keap1*; (**C**), *sod*; (**D**), *gpx*; (**E**), *cat*.

Figure 5 illustrates the impact of varying VC levels in the diet on the antioxidant nrf2 signaling pathway in the intestines of *M. salmoides* before and after heat stress. Before heat stress, different VC levels did not substantially affect the expression of nrf2 (Figure 5A), keap1 (Figure 5B), sod (Figure 5C), or gpx (Figure 5D) (*p* > 0.05). However, the expression of cat (Figure 5E) in the VC3 group was substantially higher than in the VC1 group (*p* < 0.05). After heat stress, the expression levels of nrf2 (Figure 5A), sod (Figure 5C), gpx (Figure 5D), and cat (Figure 5E) showed no significant differences between the groups (*p* > 0.05). The expression of keap1 (Figure 5B) was notably higher in the VC3 group compared to the VC1 group (*p* < 0.05). *t*-test analysis revealed that after heat stress, gpx expression (Figure 5D) was significantly higher between the groups than before heat stress (*p* < 0.05), with keap1 (Figure 5B) showing similar trends in the VC1-VC4 groups.

### 3.5. Analysis of Immune-Related Genes in the Intestine

**Figure 6:**
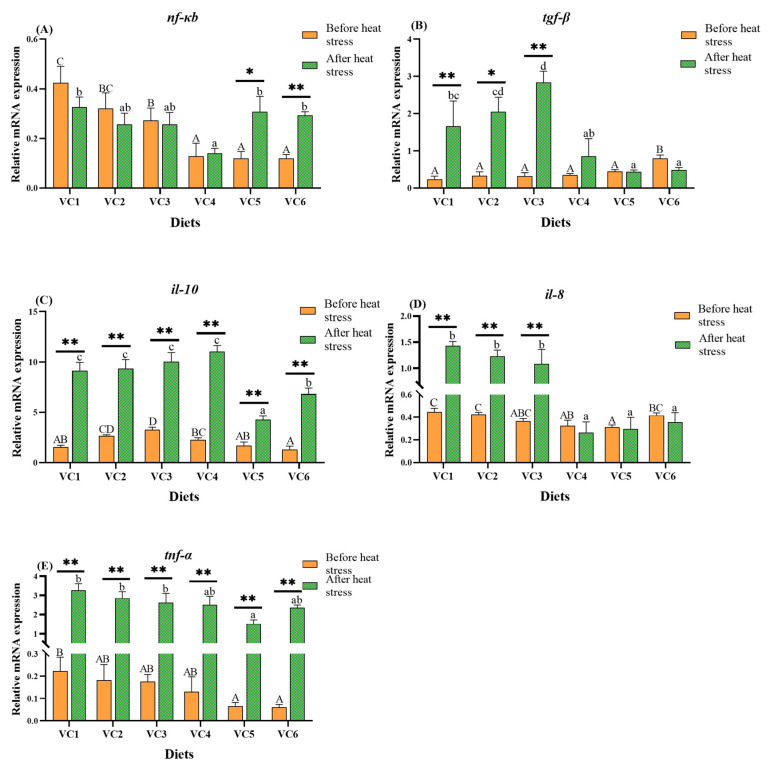
Effects of different levels of VC added to low fishmeal diets on intestinal immune-related genes in *M. salmoides* before and after heat stress. Data before heat stress are marked with uppercase letters ‘A–D’ and data after heat stress are marked with lowercase letters ‘a–d’. After *t*-test, ‘*’ for before and after heat stress data shows a statistically significant contrast (*p* < 0.05) and ‘**’ shows extremely significant (*p* < 0.01). Columns of identical color marked with different letters show significant variations between data points (*p* < 0.05). (**A**), *nf-κb*; (**B**), *tgf-β*; (**C**), *il-10*; (**D**), *il-8*; (**E**), *tnf-α*.

Figure 6 demonstrates the effects of different VC levels on antioxidant-related genes in the intestines of *M. salmoides*. Before heat stress, expressions of nf-κb (Figure 6A), il-8 (Figure 6D), and tnf-α (Figure 6E) showed a gradual decrease, being substantially lower in the VC4 and VC5 groups compared to the VC1 group (*p* < 0.05). The expression of tgf-β (Figure 6B) exhibited a gradual increase, reaching the highest level in the VC6 group (*p* < 0.05). The expression of il-10 (Figure 6C) was markedly higher in the VC3 group than in the VC1 group (*p* < 0.05). After heat stress, expressions of nf-κb (Figure 6A), il-8 (Figure 6D), and tnf-α (Figure 6E) followed a trend of initial decrease followed by an increase, with the lowest levels of nf-κb (Figure 6A) and il-8 (Figure 6D) observed in the VC4 group (*p* < 0.05), and the lowest tnf-α (Figure 6E) levels in the VC5 group (*p* < 0.05). The expression of tgf-β (Figure 6B) was significantly higher in the VC3 group (*p* < 0.05), while the expression of il-10 showed an initial increase followed by a decrease. *t*-test analysis revealed that expressions of il-10 (Figure 6C) and tnf-α (Figure 6E) were highly significant after heat stress compared to before heat stress (*p* < 0.01), while il-8 (Figure 6D) expressions also showed significant changes in the VC1-VC3 groups.

### 3.6. Expression of Apoptosis-Related Genes

**Figure 7:**
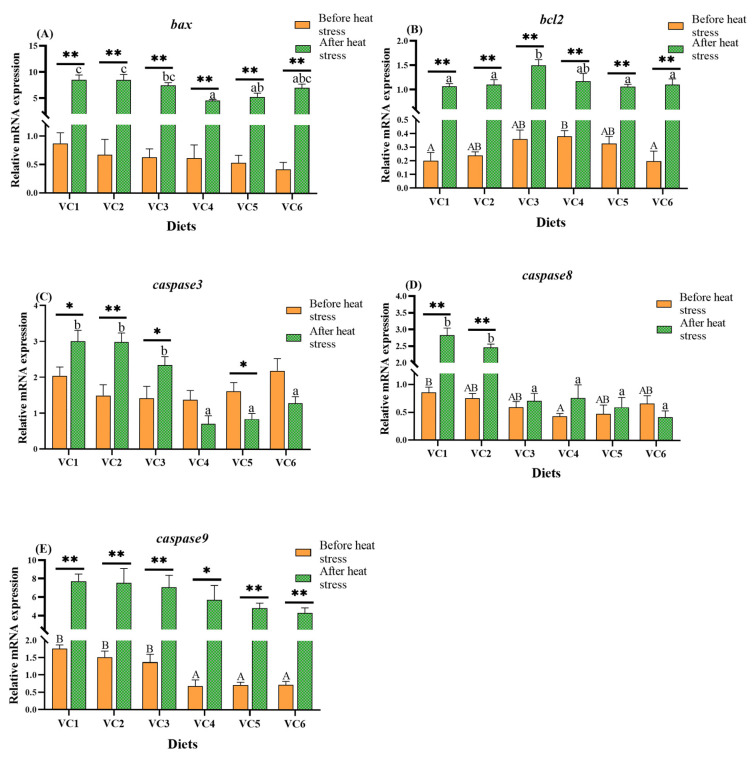
Effects of different levels of VC added to low fishmeal diets on intestinal apoptosis-related genes in *M. salmoides* before and after heat stress. Data before heat stress are marked with uppercase letters ‘A–B’ and data after heat stress are marked with lowercase letters ‘a–c’. After *t*-test, ‘*’ for before and after heat stress data shows a statistically significant contrast (*p* < 0.05) and ‘**’ shows extremely significant (*p* < 0.01). Columns of identical color marked with different letters show significant variations between data points (*p* < 0.05). (**A**), bax; (**B**), bcl-2; (**C**), caspase 3; (**D**), caspase 8; (**E**), caspase 9.

Figure 7 illustrates the effects of different VC levels in the diet on apoptosis-related gene expression in the intestines of *M. salmoides* before and after heat stress. Before heat stress, the expression of bcl-2 (Figure 7B) was significantly higher in the VC3 group compared to the other groups (*p* < 0.05). The expression levels of caspase 8 (Figure 7D) and caspase 9 (Figure 7E) were significantly lower in the VC4 group compared to the VC1 group (*p* < 0.05). No significant differences were observed in the expression of bax (Figure 7A) and caspase 3 (Figure 7C) (*p* > 0.05). After heat stress, the expressions of bax (Figure 7A), caspase 8 (Figure 7D), and caspase 3 (Figure 7C) were significantly lower in the VC4 group compared to the VC1 group (*p* < 0.05). The expression of bcl-2 (Figure 7B) initially increased and then decreased, with the highest expression observed in the VC3 group (*p* < 0.05). The expressions of caspase 3 (Figure 7C) and caspase 9 (Figure 7E) were not markedly affected by VC addition (*p* > 0.05). *t*-test analysis revealed that the expression of bax (Figure 7A), caspase 9 (Figure 7E), and bcl-2 (Figure 7B) showed highly significant differences after heat stress compared to before heat stress (*p* < 0.01).

### 3.7. TUNEL (Red) Fluorescence Analysis

**Figure 8:**
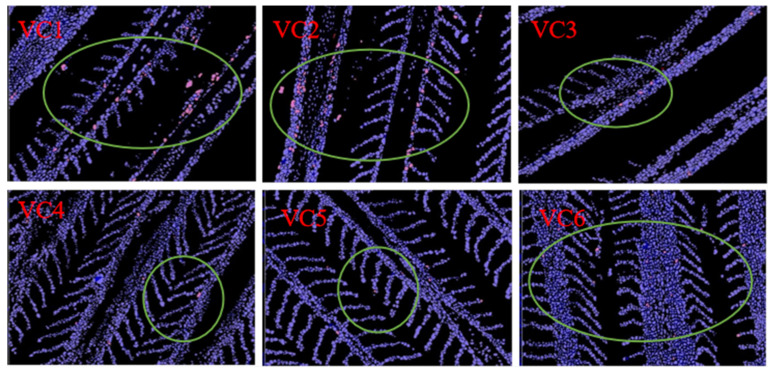
The immunofluorescence of gills of *M. salmoides* after heat stress stained with Tunel (red). The red dots in the picture represent apoptotic cells. In order to show them more easily, we labelled them using green circles.

Table 7 presents the results of TUNEL (red) fluorescence analysis of apoptosis in the gills of *M. salmoides* after heat stress, showing the effects of different VC levels. The density of red-positive cells was significantly lower in the VC3–VC5 groups compared to the VC1 group (*p* < 0.05), with the lowest density observed in the VC4 group (*p* < 0.05). The red mean density was also markedly lower in the VC4 and VC6 groups than in the VC1 group (*p* < 0.05). As shown in Figure 8, the VC3–VC5 groups exhibited the lowest number of apoptotic cells, with increased VC supplementation.

## 4. Discussion

### 4.1. Effects of VC Supplementation in Low Fishmeal Diets on the Growth Performance of M. salmoides

VC is an essential nutrient for fish growth [39]. Dietary VC supplementation has been shown to significantly enhance growth performance in species such as gibel carp (*Carassius gibelio*) [40] and Amur carp (*Cyprinus carpio haematopterus*) [41]. In this experiment, the addition of VC to the diet resulted in an initial increase in FBW, WGR, and SGR, followed by a decline. The optimal growth results were observed in the VC3 and VC4 groups. VC supplementation can enhance iron absorption [12], improve immune function [14], and contribute to collagen synthesis [42], all of which support fish growth, health, and nutrient absorption. Consistent with these findings, the addition of VC to a low-fishmeal diet improved the growth performance of juvenile *M. salmoides* in this study. Additionally, based on quadratic regression analysis of SGR and WGR, the optimal dietary VC requirement for *M. salmoides* fed low-fishmeal diets was estimated to be 0.77 g/kg. However, when VC supplementation exceeded 0.81 g/kg, a decline in growth performance was observed. This aligns with previous research on juvenile cobia (*Rachycentron canadum* L.), which showed growth inhibition when VC levels in the diet exceeded 150 mg/kg [29]. Similarly, a study on hybrid groupers (*Epinephelus fuscoguttatus*♀ × *Epinephelus lanceolatus*♂) found that excessive VC levels led to growth suppression similar to the control group [43]. High doses of VC may disrupt physiological processes, thereby slowing growth. This finding is supported by earlier studies, which suggested that VC levels above a certain threshold do not further improve growth performance [44]. Additionally, in our study, the VC supplementation did not significantly affect the whole-body composition of *M. salmoides*, which aligns with findings from research on walleye pollock (*Gadus chalcogrammus*) [45].

### 4.2. Effects of VC Supplementation in Low Fishmeal Diets on Plasma ALT and AST Levels in M. salmoides

Plasma ALT and AST levels serve as indicators of physiological changes and stress in fish, with elevated concentrations reflecting liver and tissue damage [46]. In this experiment, under low fishmeal conditions, no significant changes in ALT and AST levels were observed prior to heat stress across the different VC supplementation groups. However, after heat stress, the VC4 group showed significantly reduced ALT and AST levels compared to the VC1 group. These results align with findings from studies on coho salmon (*Oncorhynchus kisutch*) [47], juvenile Chinese sucker (*Myxocyprinus asiaticus*) [48], and juvenile striped catfish (*Pangasianodon hypophthalmus*) [49], where appropriate VC supplementation effectively reduced plasma ALT and AST levels. These observations suggest that VC supplementation in low fishmeal diets can reduce ALT and AST levels, indicating its potential to protect hepatocytes from damage. Additionally, independent sample *t*-tests revealed that ALT and AST levels were significantly higher after stress compared to before stress within the same VC supplementation groups. This indicates that the temperature conditions in the experiment likely induced oxidative stress in fish, leading to excessive ALT and AST in the blood [50]. In summary, under heat stress conditions, VC supplementation in low fishmeal diets for *M. salmoides* can reduce ALT and AST levels, thereby improving fish health.

### 4.3. Effects of VC Supplementation in Low Fishmeal Diets on Plasma and Intestinal Antioxidant Capacity of M. salmoides

Antioxidant enzyme activities and related gene expression are closely linked to tissue health [51]. *Nrf2* plays a pivotal role in antioxidant functions, regulating factors such as *sod*, *cat*, and *gpx* [52,53]. These enzymes counteract the toxicity of reactive oxygen species (ROS), while the activity of T-AOC and the levels of GSH effectively reflect the antioxidant capacity of fish [54]. In our experiment, prior to heat stress, the VC3 group exhibited higher expression levels of *cat* and greater CAT enzyme activity than the VC1 group. The downstream factors of *nrf2* and enzyme activity indicators, including T-AOC and GSH-Px, peaked in the VC3 and VC4 groups. VC supplementation in the diets of juvenile yellow catfish (*Pelteobagrus fulvidraco*) [33] and black carp (*Mylopharyngodon piceus*) [55] enhances the activities of SOD, CAT, and GSH-Px. VC also promotes *nrf2*/*keap1* signaling and gene expression, further boosting antioxidant capacity [56]. After heat stress, the VC3 group demonstrated the highest expression levels of *keap1* and *nrf2*. T-AOC and GSH levels were highest in the VC4 group, while SOD and GSH-Px levels peaked in the VC5 group. Furthermore, studies have shown that Nrf2 expression increases during stress, indicating its protective role, and it is positively correlated with the expression of antioxidant factors [57]. However, sustained elevation of Nrf2 may lead to free radical damage, apoptosis, and tumorigenesis [58,59]. In contrast, increased expression of Keap1 enhances the regulation of Nrf2 abundance, reduces its gene expression, and thereby contributes to more effective antioxidant responses [58]. Heat stress experiments on golden shiners (*Notemigonus crysoleucas*) [60] and rats [61] have shown that dietary VC enhances heat stress resistance and cellular responses. In gibel carp (*Carassius gibelio*), VC activates the *nrf2*/*keap1* pathway following acute stress, mitigating oxidative damage [40]. Before and after heat stress, MDA levels in the VC3 group were significantly lower compared to the VC1 group, indirectly indicating that adding 0.66 g/kg of VC benefits fish health. Heat stress increases mitochondrial respiration and ROS production [62]. The upregulation of genes (*keap1*, *sod*, *cat*) and the enhanced activities of antioxidant enzymes (CAT, SOD, GSH-Px) suggest that adding VC at this level improves antioxidant defenses and reduces oxidative damage induced by heat stress. Therefore, incorporating VC into low fishmeal diets enhances the antioxidant capacity of *M. salmoides* under heat stress, helping to alleviate the impacts of heat stress.

### 4.4. Effects of VC Supplementation in Low Fishmeal Diets on Intestinal Immune Function in M. salmoides

The NF-κB pathway plays a pivotal role in physiological processes such as inflammation and immune responses [63], regulating downstream immune factors [64]. VC has been demonstrated to modulate the innate immune system in fish [65]. In this experiment, the NF-κB pathway was activated before heat stress, with the expression of the anti-inflammatory factor *il-10* being highest in the VC3 group. Additionally, the expression of *tgf-β* positively correlated with VC levels, while the pro-inflammatory factors *il-8* and *tnf-α* were lowest in the VC5 group. Similar studies on juvenile grass carp [66] and yellow catfish [33] showed that VC supplementation down-regulated pro-inflammatory factors and up-regulated anti-inflammatory factors, enhancing immune capacity. Earlier research also indicated that VC enhanced immune-related gene expression in heat-stressed gibel carp, boosting lysozyme and complement C3 levels [40]. After heat stress in the present study, the NF-κB pathway remained activated, with the highest *tgf-β* expression observed in the VC3 group. The VC4 group exhibited the most reduced *il-8* levels, and the VC5 group showed the lowest *tnf-α* expression. These results suggest that VC supplementation in a low fishmeal diet activates the NF-κB pathway, promotes anti-inflammatory factor expression, inhibits pro-inflammatory factors, and protects gut health. *t*-tests revealed that after heat stress, *il-10* and *tnf-α* expressions significantly increased compared to pre-stress levels, while *tgf-β* and *il-8* expressions were most evident in the VC1-VC3 groups. Heat stress activates both innate and adaptive immune responses in fish, including the upregulation of pro-inflammatory cytokines like *il-1β*, *il-6*, and *tnf-α*, indicating an inflammatory response [67]. Thus, VC supplementation in low fishmeal diets after heat stress promotes the expression of anti-inflammatory factors, reduces pro-inflammatory responses, and enhances immune function.

### 4.5. Effects of VC Supplementation in Low Fishmeal Diets on Intestinal Apoptosis in M. salmoides

Apoptosis is regulated by the *bcl-2* family, where *bax* promotes apoptosis and *bcl-2* inhibits it [68]. *Caspase 8* and *caspase 9* require activation by *caspase 3*, and elevated mRNA levels of these *caspases* are indicative of increased apoptosis [69,70]. In this experiment, prior to heat stress, the expression of *bcl-2* was highest in the VC4 group, while *caspase 3* expression was lowest. Expression levels of *bax*, *caspase 8*, and *caspase 9* decreased with increasing VC supplementation. These results suggest that VC supplementation in a low-fishmeal diet enhances anti-apoptotic capacity, a conclusion also supported by studies on grass carp, where VC supplementation reduced pro-apoptotic genes (*bax* and *caspase 3*) and increased *bcl-2* expression [68,71]. After heat stress, the VC4 group exhibited the lowest expressions of *bax* and *caspase 8*, with *bcl-2* peaking in the VC3 group. Although changes in *caspase 3* and *caspase 9* expression were not significant, *caspase 3* expression initially decreased before increasing, while *caspase 9* expression declined with higher VC levels. Studies on gibel carp [40] and abalone [56] have shown that VC supplementation during stress reduces apoptosis. In a study on pikeperch (*Sander lucioperca*), acute heat stress significantly increased the expression of pro-apoptotic genes bax, caspase 3, and caspase 9. In this experiment, adding 0.81 g/kg VC to the diet effectively reduced apoptosis in fish [72]. Furthermore, *bax*, *bcl-2*, and *caspase 3* expressions were significantly higher after heat stress compared to before, likely due to cell damage or apoptosis induced by high temperatures [73]. Based on our findings, VC-enriched diets likely promote anti-apoptotic gene expression while reducing pro-apoptotic gene expression. These results were further confirmed by immunofluorescence experiments on gills. After TUNEL (red) staining, it was observed that VC supplementation at levels of 0.66–0.97 g/kg (VC3–VC5 groups) in low fishmeal diets effectively reduced apoptosis under heat stress conditions.

## 5. Conclusions

In conclusion, the inclusion of VC in low fishmeal diets mitigates the growth inhibition, antioxidant capacity reduction, and immune suppression associated with low fishmeal while effectively reducing apoptosis in fish. Additionally, based on quadratic regression analysis of SGR and WGR, the optimal dietary VC requirement for *M. salmoides* fed low-fishmeal diets was estimated to be 0.77 g/kg. Under heat stress conditions, VC supplementation significantly enhances the antioxidant, immune, and anti-apoptotic capacities of fish, thereby improving their resistance to acute heat stress.

## Figures and Tables

**Table 1:** Main and crude ingredients of experimental feeds.

Ingredients	VC1	VC2	VC3	VC4	VC5	VC6
Fishmeal	25	25	25	25	25	25
Chicken meal ^1^	12.00	12.00	12.00	12.00	12.00	12.00
Soy concentrated protein ^1^	13.41	13.41	13.41	13.41	13.41	13.41
Soybean meal ^1^	15.70	15.70	15.70	15.70	15.70	15.70
Wheat gluten ^1^	5.00	5.00	5.00	5.00	5.00	5.00
Blood meal ^1^	2.00	2.00	2.00	2.00	2.00	2.00
Wheat flour ^1^	8.00	8.00	8.00	8.00	8.00	8.00
Tapioca starch	5.00	5.00	5.00	5.00	5.00	5.00
Fish oil	2.80	2.80	2.80	2.80	2.80	2.80
Soybean oil	5.00	5.00	5.00	5.00	5.00	5.00
Calcium dihydrogen phosphate	3.00	3.00	3.00	3.00	3.00	3.00
Premix ^2^	2.00	2.00	2.00	2.00	2.00	2.00
Choline chloride	0.50	0.50	0.50	0.50	0.50	0.50
VC ^3^ (%)	0.00	0.05	0.10	0.15	0.20	0.25
Microcrystalline cellulose	0.25	0.20	0.15	0.10	0.05	0.00
L-Lysine ^4^ (98.5%)	0.23	0.23	0.23	0.23	0.23	0.23
L-Methionine ^4^	0.11	0.11	0.11	0.11	0.11	0.11
**Total**	100.00	100.00	100.00	100.00	100.00	100.00
**Analyzed proximate composition**						
Crude protein (%)	48.85	48.26	48.87	49.11	48.65	48.50
Crude lipid (%)	12.73	12.32	12.02	12.24	12.02	12.47
**Actual VC content (g/kg)**	0.39	0.51	0.66	0.81	0.97	1.11

^1^ Fishmeal, crude protein 65.6%, crude lipid 9.5%; Chicken meal, crude protein 62.00%, crude lipid 9.00%; Soy concentrated protein, crude protein 63.00%, crude lipid 4.10%; Soybean meal, crude protein 46.00%, crude lipid 4.25%; Wheat gluten, crude protein 80.00%, crude lipid 2.00%; Blood meal, crude protein 91.45%; Wheat flour, crude protein 13.1%, crude lipid 4.0%. They are provided from Biomar Tongwei (Wuxi) Biotech Co., Ltd. ^2^ Premixes are provided by China Wuxi Hanover Animal Health Products Co. ^3^ VC purchased from Biomar Tongwei (Wuxi) Biotech Co. ^4^ L-Lysine and L-Methionine were obtained from Feeer Co., Ltd., Shanghai, China.

**Table 2:** Formulae for calculating relevant growth properties.

Items	Full Name	Computational Formula
IBW (g)	initial body weight (g)	Total weight (g)/number of fish per bucket at the start of the culture
FBW (g)	final body weight (g)	Total weight (g)/number of fish per bucket at the end of the culture
FCR	feed conversion ratio	dry feed fed (g)/(final body weight (g) − initial body weight (g))
WGR (%)	weight gain rate (%)	100 × (final body weight (g) − initial body weight (g))/initial body weight (g)
SGR (%/day)	specific growth rate (%/day)	100 × [(Ln (final body weight (g)) − Ln(initial body weight (g)))/days]
SR (%)	survival rate (%)	100 × (survival fish number/total fish number)

**Table 3:** Reference table of experimental methods, kits and experimental equipment.

Items	Methods	Assay Kits/Testing Equipment
**Composition of diets/whole body**		
Moisture	Drying method (ID 920.36)	Electric blast drying oven (Shanghai Yiheng Scientific Instrument Co., Ltd., Shanghai, China)
Protein	Kjeldahl (ID984.13)	Auto kieldahl apparatus: Hanon K1100 (Jinan Hanon Instruments Co., Ltd., Jinan, China).
Lipid	Soxhlet (ID 991.36)	Auto fat analyzer: Hanon SOX606 (Jinan Hanon Instruments Co., Ltd., Jinan, China)
Ash	Combustion (ID 923.03)	Muffle: XL-2A (Hangzhou Zhuochi Instrument Co., Ltd., Hangzhou, China).
**Plasma parameters**		
ALT AST	International Federation of Clinical Chemistry recommended	Assay kits (ALT: 105-000442-00.AST: 15-00443-000 purchased from Mindray Medical International Ltd. (Shenzhen, China); Mindray BS 400 automatic biochemical analyzer (Mindray Medical International Ltd., Shenzhen, China).
**Plasma parameters related antioxidant capacity**		
MDA	TBA method Ammonium	
CAT	Molybdenum acid method	Assay kits (MDA: A003-1-2. CAT: A007-1-1. SOD: A001-3-2. GSH:A006-1-1. GPx: A005-1-2.T-AOC:A015-2-1) purchased from Jian Cheng Bioengineering Institute (Nanjing, China); Spectrophotometer (Thermo Fisher Multiskan GO, Shanghai, China).
SOD	WST-1 method	
GSH	Microplate method	
GSH-Px	Colorimetric method	
T-AOC	ABTS method	

**Note**: ALT, alanine aminotransferase; AST, aspartate aminotransferase. MDA, malondialdehyde; CAT, catalase; SOD, superoxide dismutase; GSH, glutathione; GSH-Px, glutathione peroxidase; T-AOC, total antioxidant capacity.

**Table 4:** All primers and internal references used in the experiment.

Genes	Forward (5′-3′)	Reverse (5′-3′)	Primer Source
*nrf2*	CACAGCAGCAGCAGGAAAAG	AAGATGCTGCCGTCTGTTGA	XM_038720536.1
*keap1*	CGTACGTCCAGGCCTTACTC	TGACGGAAATAACCCCCTGC	XP_018520553.1
*cat*	CTATGGCTCTCACACCTTC	TCCTCTACTGGCAGATTCT	MK614708.1
*sod*	GGTGTTTAAAGCCGTTTGTGTT	CCTCTGATTTCTCCTGTCACCT	XM_038708943.1
*gpx*	ATGGCTCTCATGACTGATCCAAA	GACCAACCAGGAACTTCTCAAA	XM_038697220.1
*nf-κb*	AGAAGACGACTCGGGGATGA	GCTTCTGCAGGTTCTGGTCT	XM_038699793.1
*tgf-β*	CACCAAGGAGATGCTGATT	CGTATGTTAGAGATGCTGAAG	XM_038693206.1
*il-10*	CGGCACAGAAATCCCAGAGC	CAGCAGGCTCACAAAATAAACATCT	XM_038696252.1
*tnf-α*	CTTCGTCTACAGCCAGGCATCG	TTTGGCACACCGACCTCACC	XM_038710731.1
*il-8*	GAGGGTACATGTCTGGGGGA	CCTTGAAGGTTTGTTCTTCATCGT	XM_038713529.1
*bax*	ACTTTGGATTACCTGCGGGA	TGCCAGAAATCAGGAGCAGA	Ref. [36]
*bcl-2*	TGTGGGGCTACTTTTTGGCA	TTCGACTGCCACCCCAATAC	Ref. [37]
*caspase3*	GAGGCGATGGACAAGAGTCA	CACAGACGAATGAAGCGTGG	XM_038713063.1
*caspase8*	GAGACAGACAGCAGACAACCA	TTCCATTTCAGCAAACACATC	Ref. [38]
*caspase9*	CTGGAATGCCTTCAGGAGACGGG	GGGAGGGGCAAGACAACAGGGTG	Ref. [36]
*β-action*	GGTGTGATGGTTGGTATGG	CTCGTTGTAGAAGGTGTGAT	MH018565.1

**Note:** Mean values and standard error (±SE; *n* = 3 × 3) are presented for each parameter. *nrf2*, nuclear factor erythroid 2-related factor 2; *keap1*, recombinant kelch like ech associated protein 1; *cat*, catalase; *sod*, superoxide dismutase; *gpx*, glutathione peroxidase; *nf-κb*, nuclear factor kappa-β; *tgf-β*, transforming growth factor-β; *il-10*, interleukin-10; *tnf-α*, tumor necrosis factor-α; *il-8*, interleukin-8; *bax*, bcl-2-associated x protein; *bcl-2*, b-cell lymphoma-2; *caspase3*, *caspase8* and *caspase9* belong to cysteine aspartic acid specific proteases; *β-action*, beta-actin.

**Table 5:** Impact of VC supplementation in low fishmeal feeds on *M. salmoides* growth performance.

Diets (VC mg/kg)	IBW (g)	FBW (g)	FCR	WGR(%)	SGR (%/Day)	SR (%)
VC1	2.22 ± 0.00	41.16 ± 0.20 a	0.72 ± 0.01 ^c^	1758.10 ± 8.87 a	4.87 ± 0.01 a	96.70 ± 1.67
VC2	2.22 ± 0.01	44.35 ± 0.55 b	0.69 ± 0.01 ^a^	1897.50 ± 22.25 b	4.99 ± 0.02 b	100.00 ± 0.00
VC3	2.22 ± 0.01	47.22 ± 1.14 c	0.71 ± 0.01 ^c^	2025.20 ± 46.73 c	5.09 ± 0.04 c	96.70 ± 1.67
VC4	2.22 ± 0.00	47.24 ± 0.19 c	0.72 ± 0.00 ^c^	2032.80 ± 5.97 c	5.10 ± 0.00 c	98.30 ± 1.67
VC5	2.21 ± 0.01	43.97 ± 0.48 b	0.68 ± 0.00 ^ab^	1889.40 ± 21.64 b	4.98 ± 0.02 b	100.00 ± 0.00
VC6	2.21 ± 0.00	42.90 ± 0.36 ab	0.68 ± 0.00 ^ab^	1844.10 ± 15.41 b	4.95 ± 0.01 b	98.30 ± 1.67

**Note**: Mean values and standard error (±SE) are presented for each parameter. The results of this data analysis are presented as mean ± standard error (Mean ± S.E.). Values with distinct alphabetical superscripts differ significantly (*p* < 0.05).

**Table 6:** Impact of different VC addition levels on whole fish body composition of *M. salmoides* (dry weight; %).

Diets (VC mg/kg)	Moisture (%)	Lipid (%)	Ash (%)	Protein (%)
VC1	69.58 ± 0.19	9.77 ± 0.23	3.04 ± 0.18	16.79 ± 0.19
VC2	69.48 ± 0.49	9.47 ± 0.68	3.12 ± 0.18	16.84 ± 0.19
VC3	69.35 ± 0.46	9.56 ± 0.37	3.03 ± 0.12	16.56 ± 0.09
VC4	69.17 ± 0.62	9.82 ± 0.75	3.13 ± 0.15	17.39 ± 0.35
VC5	69.35 ± 0.39	9.45 ± 0.41	3.26 ± 0.03	17.29 ± 0.48
VC6	69.49 ± 0.20	10.59 ± 0.37	3.11 ± 0.06	16.46 ± 0.25

**Note:** Mean values and standard error (±SE; *n* = 3 × 3) are presented for each parameter.

**Table 7:** Effects of different VC addition levels on apoptosis in *M. salmoides* gills after heat stress.

Diets	VC1	VC2	VC3	VC4	VC5	VC6
Red Positive Cells%	0.45 ± 0.01 ^c^	0.43 ± 0.02 ^c^	0.19 ± 0.02 ^a^	0.17 ± 0.04 ^a^	0.28 ± 0.02 ^b^	0.36 ± 0.05 ^bc^
Red Positive Cells Density, number/10^4^ pixels	0.25 ± 0.02 ^b^	0.20 ± 0.03 ^ab^	0.21 ± 0.01 ^ab^	0.17 ± 0.04 ^a^	0.19 ± 0.01 ^ab^	0.22 ± 0.00 ^ab^
Red Mean Density	0.55 ± 0.06 ^b^	0.50 ± 0.04 ^ab^	0.49 ± 0.06 ^ab^	0.37 ± 0.03 ^a^	0.44 ± 0.01 ^ab^	0.39 ± 0.03 ^a^

**Note**: Mean values and standard error (±SE; *n* = 3 × 3) are presented for each parameter. Red Positive Cells% = Red Cells Amount/Total Cells Amount; Red Positive Cells Density (number/10^4^ pixels) = Red Cells Amount/issue Area (pixel); Red Mean Density = Red Positive Density/Red Positive Area (pixel). Values with distinct alphabetical superscripts differ significantly (*p* < 0.05).

## Data Availability

Data are contained within the article.
